# Dark current modeling of thick perovskite X-ray detectors

**DOI:** 10.1007/s12200-022-00044-1

**Published:** 2022-10-31

**Authors:** Shan Zhao, Xinyuan Du, Jincong Pang, Haodi Wu, Zihao Song, Zhiping Zheng, Ling Xu, Jiang Tang, Guangda Niu

**Affiliations:** 1 Wuhan National Laboratory for Optoelectronics and School of Optical and Electronic Information, Huazhong University of Science and Technology, Wuhan, 430074 China; 2 Optical Valley Laboratory, Wuhan, 430074 China

**Keywords:** Perovskite, X-ray detection, Dark current, Semiconductor simulation, Junction device

## Abstract

Metal halide perovskites (MHPs) have demonstrated excellent performances in detection of X-rays and gamma-rays. Most studies focus on improving the sensitivity of single-pixel MHP detectors. However, little work pays attention to the dark current, which is crucial for the back-end circuit integration. Herein, the requirement of dark current is quantitatively evaluated as low as 10^−9^ A/cm^2^ for X-ray imagers integrated on pixel circuits. Moreover, through the semiconductor device analysis and simulation, we reveal that the main current compositions of thick perovskite X-ray detectors are the thermionic-emission current (*J*_T_) and the generation-recombination current (*J*_g-r_). The typical observed failures of p–n junctions in thick detectors are caused by the high generation-recombination current due to the band mismatch and interface defects. This work provides a deep insight into the design of high sensitivity and low dark current perovskite X-ray detectors.

**Graphical Abstract:**

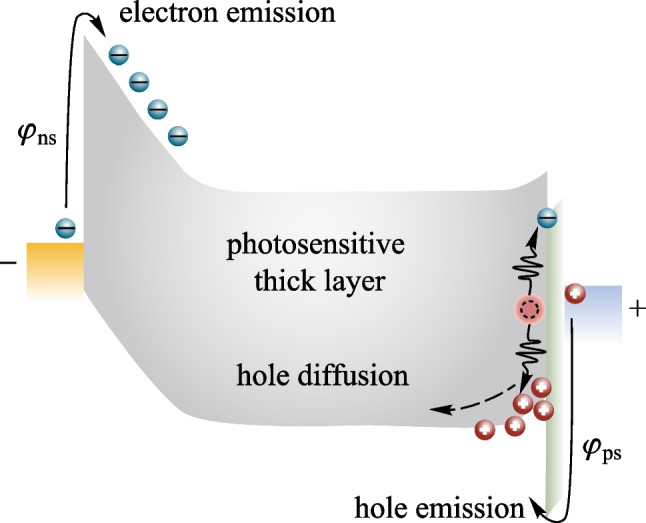

**Supplementary Information:**

The online version contains supplementary material available at 10.1007/s12200-022-00044-1.

## Introduction

Metal halide perovskites (MHPs) have attracted extensive interest in X-ray and gamma-ray detection, due to their properties such as high atomic numbers, large mobility-lifetime (*μτ*) products, defect tolerance, and low-temperature solution processability [1–4]. Many efforts have been made to improve the sensitivity of perovskite X-ray detectors, since the high sensitivity can benefit signal-to-noise ratio and reduce harmful radiation dose [5–11]. For example, the sensitivity has been rapidly increased from 80 to 23,600 μC/(Gy_air_⋅cm^2^) for MAPbBr_3_ single crystals, through strategies of crystal quality enhancement and interface engineering [5, 6, 12].

However, most of these studies focus on single-pixel devices without circuit integration. To achieve flat panel X-ray imagers (FPXIs), the semiconductor detectors should be integrated on TFT or CMOS pixel circuits. This means that the device dark current is an important figure of merit to be considered. However, previous work has paid little attention to the dark current. Currently, the dark current of perovskite X-ray detectors is in the range of 10^−8^ − 10^−5^ A/cm^2^, which is 1 to 4 orders higher than that of other semiconductor X-ray detectors such as α-Se and CdZnTe [13–15]. Moreover, the design strategy of the device structure for decreasing dark current is also not clear.

**Fig. 1:**
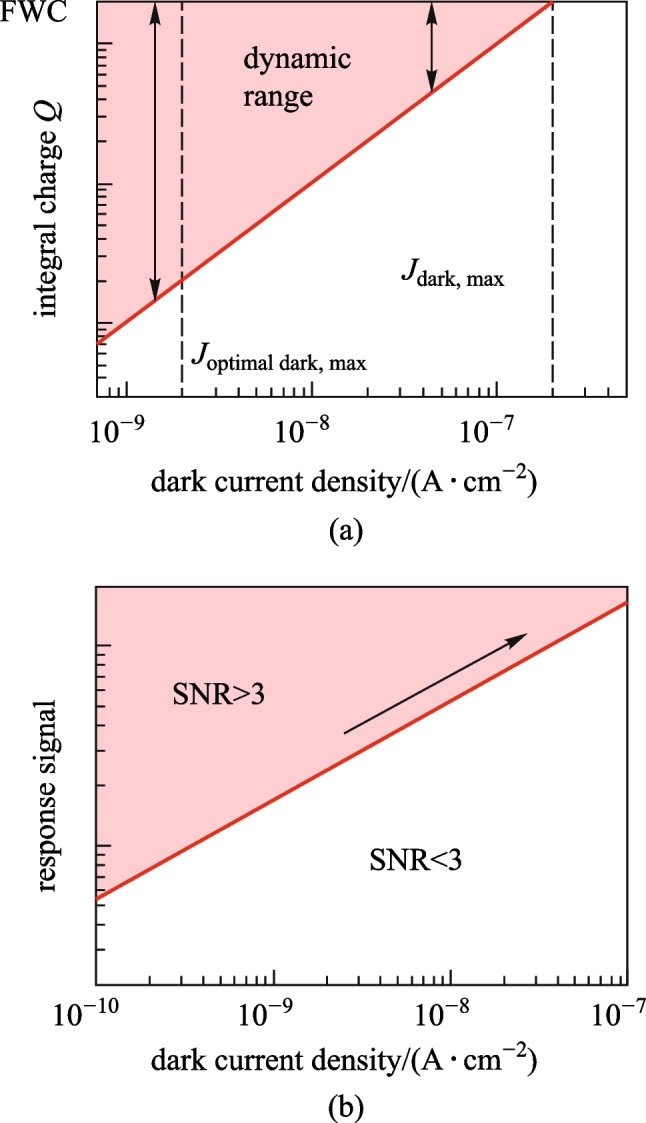
Schematic illustration of the influence of dark current in FPXIs. **a** Integral charge (*Q*) against the dark current density *J*_dark_. When *J*_dark_ flushes the collection capacitor to FWC within *t*, the dynamic range diminishes to 0. **b** Minimum detectable signal (*J*_signal, min_) against the dark current density (*J*_dark_). *J*_signal, min_ is the response signal of the minimum detection dose, corresponding to a minimum detectable signal-to-noise ratio (SNR) of 3 [33]

Herein we quantitatively evaluate the dark current requirement according to the standard process of TFT or CMOS fabs. For FPXIs, the response of the detector is firstly stored in the collection capacitor of the pixel circuit, whose capacitance is *C*_st_, and then converted to accumulated charges. When the dark current increases, the accumulated charges rise and thus reduce the dynamic response range of the imager, as shown in Fig. 1a. For pixel circuits, there is a definite threshold voltage *∆V*_max_, beyond which charges cannot be stored in the collection capacitor. According to this threshold voltage, we can derive the full well capacity (FWC) for a given pixel circuit as FWC = *C*_st_ × *∆V*_max_. Then we can obtain the threshold of the response current density (*J*_max_), beyond which the circuit cannot differentiate the intensities of responses, as *J*_max_ = (*C*_st_ × *∆V*_max_)/(*A* × *t*), where *A* is the pixel area, *t* is the integration time. For a typical dynamic FPXI (frame rate: 30 fps), *A* is 150 × 150 μm^2^, *t* is 33 ms, *C*_st_ is ~ 1 pF for a 150 × 150 μm^2^ pixel, and *∆V*_max_ is set as 1.5 V [16]. Then *J*_max_ can be estimated as ~ 2 × 10^−7^ A/cm^2^. The dark signal should be limited to a maximum of 1% of *J*_max_ [17], i.e., ~ 10^−9^ A/cm^2^, to guarantee the response dynamic range.

Moreover, the dark current can also cause noise fluctuations. The detector noise is mainly composed of thermal noise, shot noise, and 1/*f* noise [18]. The 1/*f* noise decays rapidly with frequency and can be eliminated by interface optimization. The thermal noise (*I*_n,t_) and shot noise (*I*_n_) are defined as ${I}_{\mathrm{n},\mathrm{ t}}=\sqrt{4kT\Delta f/R}$ and ${I}_{\mathrm{n}}=\sqrt{2e{\Delta f\cdot I}_{\mathrm{dark}}}$, respectively, where *R* is the bulk resistance of the detector, *k* is the Boltzmann constant, *T* is the temperature, *I*_dark_ is the dark current, and Δ*f* is the bandwidth in the frequency domain. For the typical photo-conducting detectors, it is clear that the dark current directly or indirectly aggravates the noise signal. The signal-to-noise ratio of the X-ray flat plate detector is increased by noise enhancement, as shown in Fig. 1b.

To decrease the dark current, the bulk resistance of perovskite semiconductors should be enhanced, which is inversely proportional to the carrier concentration and mobility. Many efforts have been devoted to decreasing the carrier concentration and approaching the intrinsic semiconducting property [7, 11, 19, 20], but only a few cases have succeeded. The study of solar cells, LEDs and photodetectors has shown that Schottky junction and p–n junction type devices are effective in suppressing the dark current [21, 23]. It should be noted that all the above-mentioned devices are based on thin films, where the thickness of the photosensitive layer is hundreds of nanometers for solar cells and photodetectors, and < 100 nm for LEDs [24]. However, for X-ray detectors, the thickness of the photosensitive layer should be hundreds of micrometers, considering the high penetration capability of X-rays [1]. For thick X-ray or gamma-ray  perovskite detectors, the rectification ratios of junction devices are generally poor, especially for p–n junctions (Table 1). The underlying mechanism is still not clear.

**Table 1:** Summary of the junction structures and rectification ratios of MHP polycrystalline thick film or bulk single crystal detection devices

Material	Perovskite morphology	Device structure	Structure type	Rectification ratio
MAPbI_3_ [25]	Single crystal	Ga/MAPbI_3_/Au	Schottky junction	1100 (@ ± 20 V)
CsPbBr_3_ [26]	Single crystal	Ga/CsPbBr_3_/Au	Schottky junction	962 (@ ± 35 V)
CsPbBr_3_ [7]	Polycrystalline film	Au/CsPbBr_3_/FTO	Schottky junction	21 (@ ± 1.5 V)
CsPbBr_3_ [27]	Single crystal	EGaIn/CsPbBr_3_/Au	Schottky junction	550 (@ ± 200 V/cm)
		Ga/CsPbBr_3_/PTAA/Au	Schottky junction	660 (@ ± 100 V/cm)
		Ag/C_60_/CsPbBr_3_/PTAA/Au	p–n junction	1.0 (@ ± 110 V/cm)
MAPbBr_3_ [12]	Single crystal	Au/MAPbBr_3_/C_60_/BCP/Ag	p–n junction	1.1 (@ ± 0.5 V)
MAPbBr_3_ [28]	Polycrystalline polymer film	Cu/BCP/C_60_/MAPbBr_3_&PMMA/NiO_*x*_/ITO	p–n junction	1.0 (@ ± 1.0 V)
MAPbBr_3_ [29]	Single crystal	Cr/MAPbBr_3_/C_60_/BCP/Cr	p–n junction	3.3 (@ ± 5.0 V)
MAPbBr_3_ [6]	Single crystal	Au/poly-TPD/MAPbBr_3_/C_60_ /PCBM/Ag	p–n junction	1730 (@ ± 29 V)
MAPbI_3_ [30]	Polycrystalline film	ITO/TiO_2_/MAPbI_3_/Spiro-MeOTAD/ITO	p–n junction	1.6 (@ ± 40 V)
Cs_0.15_FA_0.85_PbI_3_ [31]	Polycrystalline filled film	C/Cs_0.15_FA_0.85_PbI_3_/C_60_/BCP/Cr	p–n junction	1.3 (@ ± 25 V)
Cs_2_TeI_6_ [32]	Polycrystalline film	Au/PTAA/Cs_2_TeI_6_/TiO_2_/FTO	p–n junction	1.4 (@ ± 0.5 V)

In this work, through semiconductor device analysis and simulation, we reveal that the main current compositions of junction devices are the thermionic-emission current (*J*_T_) and the generation-recombination current (*J*_g-r_). The junction can effectively suppress *J*_T_ by constructing the carrier blocking barrier. The low barrier at the p- or n-layer is one reason for the poor performance of thick p–n junction detectors. In addition, non-ideal *J*_g-r_ is another critical issue degrading the performance of devices. Both heterojunction band mismatches and traps within bulks or interfaces are the cause of *J*_g-r_. We determine experimentally that the interfacial defects are prone to cause the failure to form a junction region between the thick perovskite layer and thin p- or n-layer. We believe that the key to obtaining excellent thick detectors is the fabrication of p–n junctions with band match and without introducing interface defects.

## Theoretical dark current modeling of thick devices

**Fig. 2:**
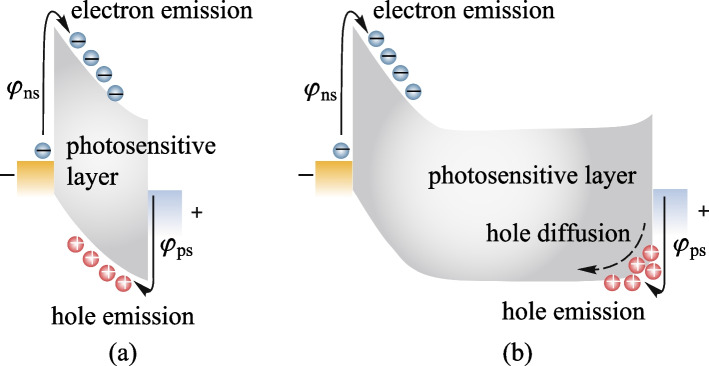
Energy band diagrams of the Metal-Semiconductor-Metal (MSM) device with **a** thin and **b** thick photosensitive layers

Figures 2a, b exhibit energy band diagrams of the basic Metal-Semiconductor-Metal (MSM) device with thin and thick photosensitive layers. Theoretically, two depletion regions are formed at the two sides of the device due to the Schottky barriers between the electrode and the high-resistivity perovskite. If the photosensitive layer thickness is less than the depletion region width, as shown in Fig. 2a, the junction regions are punched through, which is a full depletion case [34]. The depletion region width *X*_d_ can be approximately calculated as Eq. (1) [35]

1 $${X}_{\mathrm{d}}={\left[\frac{2\varepsilon ({V}_{\mathrm{D}}-V)}{q{N}_{\mathrm{d}}}\right]}^{1/2},$$

where *q* is the electron charge, *ε* is the dielectric constant, *N*_d_ is the doping concentration of the semiconductor, *V* is the operating voltage, and *V*_D_ is the barrier height, i.e., ${V}_{\mathrm{D}}={W}_{\mathrm{m}}-{W}_{\mathrm{s}}$. *W*_m_ and *W*_s_ are the work function of metal electrode and the semiconductor, respectively. For MHPs, *X*_d_ is approximately 10 μm for barrier height of 1 V, where *ε* is 10*ε*_0_ and *N*_d_ is 10^14^ cm^−3^ for MAPbI_3_ polycrystalline films [36, 37]. For thin-film detectors with thickness of hundreds of nanometers, *X*_d_ is much larger than the layer thickness *L* and the device is fully depleted. For thick X-ray detectors, the thick layer makes it difficult to obtain a fully depleted device. Assuming *X*_d_ = *L* = 200 μm, the punch-through voltage *V*_PT_ is calculated as ~ 4000 V using Eq. (1). The neutral region cannot effectively collect generated charges due to the absence of electric field [35]. Therefore, it is necessary to reduce the thickness or enhance the operating voltage to achieve full depletion and obtain a high detection efficiency.

On the other hand, theoretically, incomplete depletion would not degrade the dark performance of the junction device. For the Schottky contact where the depletion region is the barrier layer, the dark current is determined by carrier injection, following the thermionic-emission theory as represented by Eq. (2) [38]

2 $${J}_{\text{T}}={A}^{*}\cdot {T}^{2}\cdot {\mathrm{e}}^{-\frac{q{\varphi }_{\mathrm{s}}}{kT}}\cdot \left({\mathrm{e}}^\frac{qV}{kT}-1\right),$$

where A* is Richardson constant, which is 120 A/(cm^2^⋅K^2^) for vacuum, *qφ*_s_ is the barrier height of carrier injection. The reverse saturation dark current is only related to *φ*_s_. When the carrier injection barrier is higher than 1 eV, the theoretical dark current is lower than 2 × 10^−10^ A/cm^2^. For Ohmic-like contact where the depletion region is the accumulation layer, the current source is a synthesis of carrier thermionic-emission and diffusion process [35]. Injected carriers are accumulated in the depletion region, and this impedes the flow of carriers, which then relies on diffusion movement to enter the semiconductor interior. Correspondingly, the diffusion current *J*_D_ is lower than *J*_T_ for the same barrier. Hence as long as sufficiently high barriers are constructed on both sides of contacts, fully depleted thin- and non-fully depleted thick-film devices both can realize theoretically low dark currents. The p–n junction device can construct higher barriers than that of the Schottky junction by introducing the blocking layer (BL), thus to more effectively suppress the dark current.

The generation-recombination current (*J*_g-r_) is another crucial source of the dark current, which is the main cause for deterioration of the *J–V* characteristic of the device. *J*_g-r_ is contributed by the bulk depletion region and the interface. For the bulk layer, according to the Shockley–Read–Hall (SRH) recombination model, *J*_g-r_ can be calculated using Eq. (3) [39]

3 $${J}_\text{g-r}=\frac{q{n}_{\mathrm{i}}{X}_{d}}{2\tau }\left({\mathrm{e}}^\frac{qV}{2kT}-1\right),$$

wh﻿ere *n*_i_ is the intrinsic carrier concentration, and *τ* is the non-equilibrium carrier lifetime, which is inversely proportional to the trap concentration *N*_t_. *J*_g-r_ is proportional to *N*_t_ and *X*_d_, and cannot be suppressed by the barrier. Since the thickness of the X-ray detectors is as large as tens to hundreds of microns, *J*_g-r_ is significantly raised with the increase of the perovskite trap concentration.

As shown in Additional file 1: Fig. S1, the interface defects and the energy band offset of the p–n heterojunction would lower the thermionic emission barrier *qφ*_s_, and facilitate undesired charge generation at the interface [40]. Therefore, in order to obtain high-performance thick junction devices, in addition to building a high barrier by the BL to reduce *J*_T_, the photosensitive layer should be synthesized with few bulk and interface traps, and the BL should maximize *φ*_s_ with matched *E*_c_ or *E*_v_ to reduce *J*_g-r_. Currently, the performance of p–n junction devices is inferior to that of a Schottky junction, which is to be expected due to the combined result of unsuitable BL (with low blocking barriers) and interfacial defects.

## Simulation modeling and parameters

To further verify the above analysis, the X-ray detectors were simulated by Solar Cell Capacitance Simulator (SCAPS) software. MAPbI_3_ was selected as the photosensitive layer of the detector. The material parameters are listed in Table 2. Two types of defects in the bulk layer were set, containing shallow donor and deep neutral defects. The shallow donor density was set as 1 × 10^14^ cm^−3^. The deep defects were located at the most effective level as the band gap center (*E*_i_), whose density was 10^12^ cm^−3^ [37].

**Table 2:** Details of basic parameters used for the simulation of MAPbI_3_ and SnO_2_ layer

Parameters	MAPbI_3_	SnO_2_
Thickness/μm	0.8/200	0.1
Band-gap/eV	1.5 [42]	3.6 [45]
Electron affinity/eV	3.9 [42]	4.0 [45]
Dielectric permittivity (relative)	30 [43]	9 [45]
CB effective density of states/cm^−3^	2.5 × 10^20^ [43]	2.2 × 10^18^ [45]
VB effective density of states/cm^−3^	2.5 × 10^20^ [43]	1.8 × 10^19^ [45]
Electron thermal velocity/(cm⋅s^−1^)	1 × 10^7^	1 × 10^7^
Hole thermal velocity/(cm⋅s^−1^)	1 × 10^7^	1 × 10^7^
Electron mobility/(cm^2^⋅V^−1^⋅s^−1^)	10 [44]	100 [45]
Hole mobility/(cm^2^⋅V^−1^⋅s^−1^)	10 [44]	25 [45]
Donor density/cm^−3^	1 × 10^14^ [37]	1 × 10^16^/0
Acceptor density/cm^−3^	0	0/1 × 10^16^
Defect type	Neutral	Neutral
Capture cross section of electrons/cm^2^	1 × 10^–14^	1 × 10^–14^
Capture cross section of holes/cm^2^	1 × 10^–13^	1 × 10^–14^
Defect energy level above *E*_i_/eV	0	0
Defect density/cm^−3^	1 × 10^12^ [37] (1 × 10^10^ − 1 × 10^15^)	1 × 10^14^

We designed Schottky junctions with various contact electrodes and p–n junction with SnO_2_ layer, respectively. The Schottky junction device was composed of ITO/MAPbI_3_/Au or Ga, while the structure of the p–n junction device was ITO/SnO_2_/MAPbI_3_/Au or Ga. SnO_2_ has been widely used as the electron transport layer in p–i–n perovskite solar cells [41]. The donor density of SnO_2_ was set as 1 × 10^16^ cm^−3^. The parameters of contact electrodes are listed in Table 3. The back metal was a Schottky contact electrode such as Au (5.1 eV) and Ga (4.2 eV). Gold electrodes are widely used for perovskite radiation detectors, while Ga has been studied for Schottky contact of CsPbBr_3_ single crystal [26].

**Table 3:** Material parameters of the contact electrodes

Parameters	Au	ITO	Ga
Electron recombination velocity/(cm⋅s^−1^)	1 × 10^5^	1 × 10^7^	1 × 10^5^
Hole recombination velocity/(cm⋅s^−1^)	1 × 10^7^	1 × 10^5^	1 × 10^7^
Metal work function/eV	5.1	4.7	4.2
Allow contact tunneling	On	On	On

## Results and discussion

**Fig. 3:**
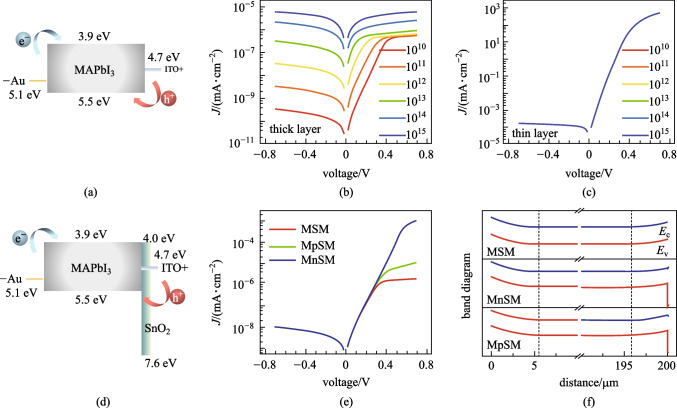
Simulated structures and output performances of MAPbI_3_ detectors. **a, d** Band diagram of MAPbI_3_ devices with  **(a)** the Schottky junction and **(d)** the p–n junction. **b**, **c** are the simulated *J*–*V* curves of thick layer and thin layer devices based on the structure (**a**), with different deep defect concentrations. **e** Comparison between the simulated *J*–*V* characteristics with Schottky, n-type and p-type junction, where M denotes Au or ITO electrode, S denotes MAPbI_3_ semiconductor, and n or p denotes n-type or p-type SnO_2_ layer. **f** Simulated energy band of devices with Schottky, n-type and p-type junction

As shown in Fig. 3a, the Schottky device with ITO/MAPbI_3_/Au structure was simulated to analyze the dark current source. The bias voltage was applied to the Au electrode. The thicknesses of MAPbI_3_ were 200 and 0.8 μm, corresponding to thick X-ray detectors and thin-film photodetectors. We set a range of deep defect concentrations *N*_t_ of the perovskite layer from 10^10^ to 10^15^ cm^−3^, corresponding to high-quality single crystals and polycrystalline films [37]. The simulated current density–voltage (*J*–*V*) characteristic curves of the two devices with different thicknesses were obtained as shown in Fig. 3b, c. With a negative bias on the Au contact, the high electron-injection barrier of 1.2 eV could lead to a low reverse current. For the thin-film device, no difference in the *J*–*V* curves was found for various defect concentrations. The reverse currents of the thin device all were 1.7 × 10^–7^ A/cm^2^ at − 0.7 V. This thin device was fully depleted as shown in Additional file 1: Fig. S2. The theoretical dark current density was dominated by *J*_T_, which could be calculated by Eq. (2) as the sum of 3 × 10^–14^ A/cm^2^ (for electrons with a 1.2 eV barrier) and 1 × 10^–7^ A/cm^2^ (for holes with a 0.8 eV barrier). The estimated *J*_T_ was consistent with the simulation results of the current for the thin-film device. In Additional file 1: Fig. S3, *J*_g-r_ of this device was shown to have changed from 5 × 10^–15^ to 5 × 10^–10^ A/cm^2^ with the increase of *N*_t_, which could be negligible compared with *J*_T_. For the thick device, the current rose from 3.4 × 10^–13^ to 6.1 × 10^–9^ A/cm^2^ at − 0.7 V bias with the increase of *N*_t_. Simultaneously, the rectification ratio decreased to 1 at ± 0.7 V. This was due to *J*_T_ of holes being suppressed by the accumulation region on ITO side, and *J*_g-r_ became an important current composition. In this case, the quality of photosensitive material significantly influenced the performance of the thick junction detector.

Furthermore, we simulated the p–n junction device of perovskite X-ray detectors. Figure 3d showed the energy band of ITO/SnO_2_/MAPbI_3_/Au junction device. SnO_2_ layer served as a high hole-injection barrier of 2.9 eV. In Fig. 3e, whether SnO_2_ layer is n-type or p-type semiconductor, the rectifying junction was oriented in the same direction. Figure 3f can confirm that the band bending of the MAPbI_3_ depletion region was almost unchanged whether adding SnO_2_ layer or not. The widths of the depletion region were the same, which were 5.5 μm at Au contact and 4.2 μm at ITO contact. Consequently, we consider that the transport layer of p–n junction in thick devices hardly influences the depletion region and it acts as a charge blocking layer. In addition, Fig. 3e showed that the current densities for devices with Schottky, n-type and p-type junction were all 1.0 × 10^–11^ A/cm^2^ at − 0.7 V, because SnO_2_ only blocks the injection of holes which hardly contributes to the dark current. SnO_2_ layer is hole-blocking by virtue of the matched conduction band and deep valence band. Its carrier type is not critical for the reverse current of the thick device.

**Fig. 4:**
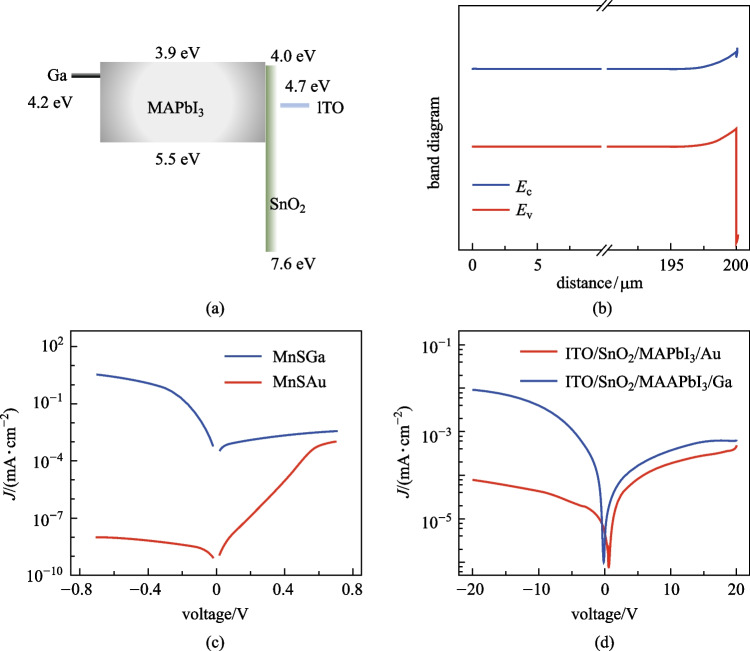
Simulated and experimental characteristics of Schottky devices. **a** Band diagram of the junction device with Ga electrode. **b** Simulated energy band of the junction device with Ga electrode. Comparison of the **c** simulated and **d** experimental *J*–*V* curves with Au and Ga metal contacts

We studied the influence of Schottky barrier for X-ray thick detectors using two metal–semiconductor contacts of Ga/MAPbI_3_ and Au/MAPbI_3_. The device structure with the Ga electrode was shown in Fig. 4a. The bias voltage was applied to the Ga electrode. The work function of Ga metal (as 4.2 eV) was matched with the fermi level of MAPbI_3_ (as 4.198 eV by simulation), so the Ga electrode was an ohmic contact. Figure 4b displays the simulated energy band of this device, showing the band on the Ga side was flat and the junction region was on the SnO_2_/ITO side. In Fig. 4c, since the barrier *V*_D_ of Ga electrode (0.002 eV) was much lower than that of Au electrode (0.9 eV), *J* for the device with Ga electrode (3.4 × 10^–3^ A/cm^2^) was 8 orders higher than that with Au electrode at − 0.7 V bias. This effectively illustrates that the high Schottky barrier played an important role in blocking carrier flow and reducing the dark current.

Following the simulated structure as mentioned above, we fabricated the X-ray detector employing perovskite. We used the process of one-step blade-coating to fabricate MAPbI_3_ polycrystalline thick films. This method has the advantages of easy preparation of functional layers, large area, and easy integration at low temperature, which is compatible with the processing of TFT circuits [30, 46]. A detailed description of the fabrication is provided in the supplementary Additional file 1: “Experimental Section”. The *J–V* curves of the experimental detector are shown in Fig. 4d. Under − 20 V bias, *J* for the case of Ga electrode (9.2 × 10^–6^ A/cm^2^) was higher than that of Au electrode (7.6 × 10^–8^ A/cm^2^). The device rectification was similar to the simulation result, indicating the validity of Schottky junction in practice. In previous studies, the Schottky device structure has been applied in X-ray/gamma-ray detectors of perovskite single crystal or polycrystalline films, and has been confirmed to be beneficial for obtaining steady low dark currents [25, 27].

**Fig. 5:**
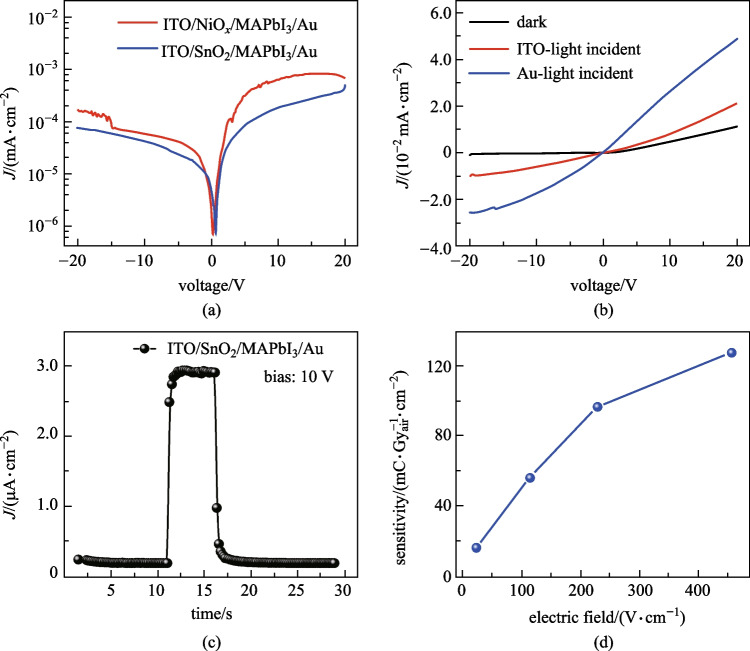
Detection performance of MAPbI_3_ thick films. **a** *J−V* curves of the X-ray detectors with SnO_2_ and NiO_*x*_ layer. **b** Visible light response-voltage characteristics at both surfaces for the ITO/SnO_2_/MAPbI_3_/Au device. **c** X-ray response, with the electric field set as 238 V/cm. **d** Sensitivity as a function of the electric field

Although the experimental device of ITO/SnO_2_/MAPbI_3_/Au structure exhibits *J−V* characteristic similar to the simulation result, the rectification ratio is just 5.6 at ± 20 V which is 4 orders lower than the simulated theoretical value (~ 10^5^ at ± 0.7 V). In the simulation, the interface was considered to be a perfect contact and interfacial defects were ignored. However, interfacial defects were believed to be an important reason for deteriorating perovskite detectors. Here we prepared two kinds of blocking layers, one of SnO_2_ and the other of NiO_*x*_, to analyze the influence of the bottom interface of thick perovskite films. The SnO_2_ layer was fabricated by spinning coating the colloidal dispersion, and NiO_*x*_ was deposited by radio frequency (RF) sputtering. The band structure of the device with NiO_*x*_ layer is shown in Additional file 1: Fig. S4a [47]. NiO_*x*_ acted as an electron blocking layer, with an electron injection barrier of 2.9 eV. Theoretically, the device with NiO_*x*_ is reverse-biased under positive voltage (where electrons are injected from ITO), while the device with SnO_2_ layer is reverse-biased under negative voltage. This can be corroborated by the simulated *J−V* curve in Additional file 1: Fig. S4b. Figure 5a shows the *J−V* curves of the experimental devices with SnO_2_ and NiO_*x*_ layer. The devices showed similar rectification characteristics, with rectification ratios of 5.6 and 4.7 at ± 20 V, respectively. The results indicated that the bottom interface of thick perovskite films did not form a junction with the blocking layer. High concentrations of interfacial defects and traps are considered as the reason for the failure of the p–n junction. These interfacial trapped charges cause surface *J*_g-r_, which results in the leakage current that cannot be suppressed by the reverse bias.

To further determine the interface quality, we tested the response signal of 532 nm light incident from both electrodes. The visible light mainly deposited its energy on the incident surface (~ 500 nm) of the thick film. The distances between the light source and the device surfaces were both 10 cm, to ensure the same incident optical power. As shown in Fig. 5b, when light was incident from ITO, the photocurrent (9.0 × 10^–6^ A/cm^2^ operating at − 20 V) was lower than that from Au (2.5 × 10^–5^ A/cm^2^ at − 20 V). The transmittance of Au layer to 532 nm wavelength light was approximately one-fourth of that of ITO, and the SnO_2_ layer was almost completely transparent for 532 nm light (Additional file 1: Fig. S5). Neglecting the light leaking from the edge of the Au electrode, the photo response on MAPbI_3_ bottom interface was reduced by about 90% at − 20 V. The loss was caused by the nonradiative recombination of interface traps, which demonstrated that the quality of the polycrystalline perovskite thick-film bottom was poor. There is an urgent need for more efficient surface-interface engineering for perovskite thick devices.

Finally, we exposed the X-ray detector with the structure of ITO/SnO_2_/MAPbI_3_/Au to an X-ray source (VARIAN RAD-14) with a 50 kV operation voltage and 25 mA operation current. Attenuated through a 1 mm aluminum plate and a 0.3 mm copper plate, the X-ray beam quality was standard RQA3 and the dose rate was 28.32 μGy_air_/s. The thickness of the MAPbI_3_ and SnO_2_ film was 438 μm and ~ 100 nm (Additional file 1: Fig. S6) respectively. The current density curve of the thick detector is provided in Fig. 5c. Operating at − 10 V, the light–dark ratio of X-ray response was 14.5 with a stable dark current density of 2.0 × 10^–7^ A/cm^2^. This dark current was on par with the reported performance of MAPbI_3_ thick-film Schottky device (~ 1.2 × 10^–7^ A/cm^2^ under the same electric field) [48]. As mentioned above, the large dark current was due to the poor quality of the bottom interface resulting in a high *J*_g-r_. In addition, there was a lack of a matched electron blocking layer between the perovskite film and the Au electrode to further suppress the current. Figure 5d shows the sensitivities of the thick device versus applied voltages. This X-ray detector exhibited a high sensitivity of 16,300 μC/(Gy_air_⋅cm^2^) at 23 V/cm, which is three orders of magnitude higher than α-Se X-ray detectors (20 μC/(Gy_air_⋅cm^2^)), and is high enough for the application of FPXIs [14, 49]. The nonlinear increase of sensitivity with the electric field indicates that the device had high photoelectric gain caused by interfacial and grain boundary defects, which impaired the linear dynamic range of FPXIs. The processing of perovskite thick-film devices needs to be optimized to eliminate this nonlinearity.

## Conclusion

In summary, this paper proposes that dark current is an important issue affecting integration of MHP X-ray detectors with pixel circuits. The requirement of dark current is evaluated as 10^–9^ A/cm^2^ for FPXIs. To realize this low dark current, it is necessary to redesign the junction device. We reveal the source of the dark current for thick MHP junction detectors as the thermionic-emission current and the generation-recombination current. The former is suppressed by the blocking barrier. The latter is the main reason for the poor performance of thick MHP p–n junctions, which is caused by hetero-band mismatch and interface defects. Due to the interface defect issue, the fabrication of the p–n junction is prone to failure. The related surface-interface engineering and processing need to be intensively studied. Overall, our work provides progress towards optimization of the performance of junction devices for perovskite X-ray detectors applying to FPXIs.

## Supplementary Information

Below is the link to the electronic supplementary material.

**Additional file 1. **Experimental Section and other supplementary materials including Figs. S1 to S6.
