# Supplementary material for: Dark current modeling of thick perovskite X-ray detectors
Source: Front Optoelectron. 2022 Oct 31;15(1):43. doi: 10.1007/s12200-022-00044-1 (PMC9756221; doi:10.1007/s12200-022-00044-1)
Supplement: Supplementary file 1 — Additional file 1. Experimental Section and other supplementary materials including Figs. S1 to S6. [file 12200_2022_44_MOESM1_ESM.pdf]

Additional file 1 for

Dark current modeling of thick perovskite X-ray detectors

Shan Zhao[1], Xinyuan Du[1], Jincong Pang[1], Haodi Wu[1], Zihao Song[1], Zhiping Zheng[1,2], Ling Xu[1,2], Jiang Tang[1,2], Guangda Niu[1,2]

1. Wuhan National Laboratory for Optoelectronics and School of Optical and Electronic Information, Huazhong University of Science and Technology, Wuhan 430074, China.
2. Optical Valley Laboratory, Wuhan 430074, China.

Email: guangda_niu@hust.edu.cn

Additional file 1: Experimental Section

Materials. Lead iodide (PbI_2) (99.999%), methylammonium iodide (CH_3NH_3I) (MAI, 99.9%), and the ITO on glass were purchased from Advanced Election Technology Co. Ltd. Anhydrous γ-butyrolactone (GBL) was obtained from Aladdin Chemical Co. Ltd. Tin(IV) oxide, 15% in H_2O colloidal dispersion was purchased from Alfa Aesar. Target materials NiO for magnetron sputtering and Au grains were obtained from ZhongNuo Advanced Material Technology Co., Ltd. Ga metallic liquid or solid (99.99%) was purchased from Sigma-Aldrich. All chemicals were used without further purification.

Fabrication of the transport layer. The colloidal dispersion Tin(IV) oxide was diluted and dispersed with water in a ratio of 1:2 under 10 min hyperacoustic vibration. The ITO substrates were treated by 10 min ozone plasma to be hydrophilic after a standard cleaning process. Next, the as-prepared Tin oxide solutions were coated onto the ITO substrate by a spin-coating process at 3000 rpm for 30 s. Then the obtained samples were thermally annealed on a hot plate at 150°C for 30 min. 50 nm NiO_x layer was deposited on the ITO glass by radio frequency (RF) sputtering, where the flow rate of argon was set at 100, the power was set at 200 W and the duration was 7 minutes with rotation to ensure uniformity.

Fabrication of the $MAPbI_3$ thick film. Equivalent molar ratios of MAI and PbI_2 raw materials were dissolved in GBL under magnetic stirring at 60 °C until forming a homogeneous solution (4.5 M). The final viscous solution was coated on SnO_2 or NiO_x substrate by a blade. The film thickness was controlled by the distance (800 μm) between the blade and substrate. The coated films were annealed on a hot plate at 80 °C for 12 h for complete GBL removal. Finally, 60-nm thick Au electrode was evaporated on the top surface, or liquid metal Ga was coated on the surface.

Detector performance measurement. For X-ray detection performance, VARIAN RAD-14 tube was used as the X-ray source. Attenuated through a 1 mm aluminum plate and a 0.3mm copper plate, the X-ray beam quality is standard RQA3 and the dose rate is

28.32 μGy_{air} s^{-1}. The dose rate had been calibrated with the ion chamber dosimeter (Magic Max from IBA Dosimetry). For visible light response performance, the light source was 532-nm LED illumination. We used Keithley 6571B Source Meter to apply bias voltages and record the response current. All measurement about detection was conducted in a dark lead box to minimize interference from ambient light and noise response.

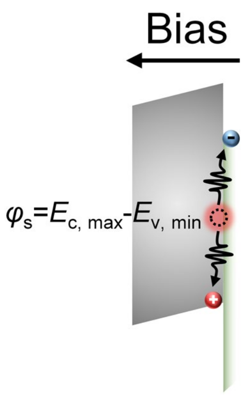

Additional file 1: Fig.S1 Schematic diagram of the thermionic generation mechanism at the HBL-perovskite interface.

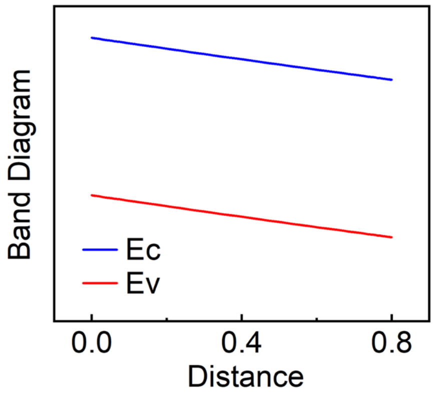

Additional file 1: Fig.S2 The band diagram of the Au/$MAPbI_3$/ITO device with the thickness of perovskite as 0.8 μm.

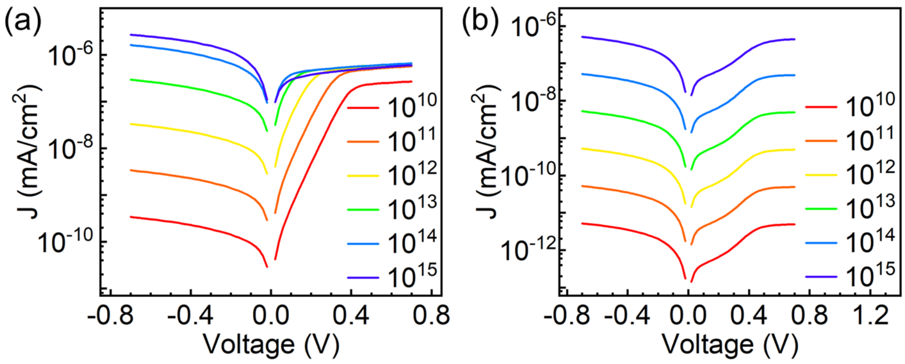

Additional file 1: Fig.S3 The simulated generation-recombination current curves of the thick device (a) and the thin device (b) with different trap concentrations.

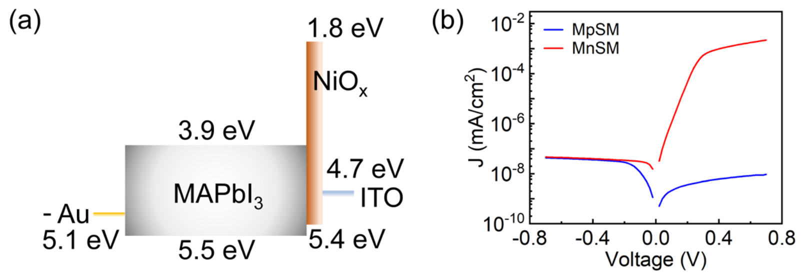

Additional file 1: Fig.S4 (a) The band structure of the ITO/NiO_x/$MAPbI_3$/Au device. (b) The simulated *J-V* curve of ITO/SnO_2/$MAPbI_3$/Au (MnSM) and ITO/NiO_x/$MAPbI_3$/Au (MpSM) device.

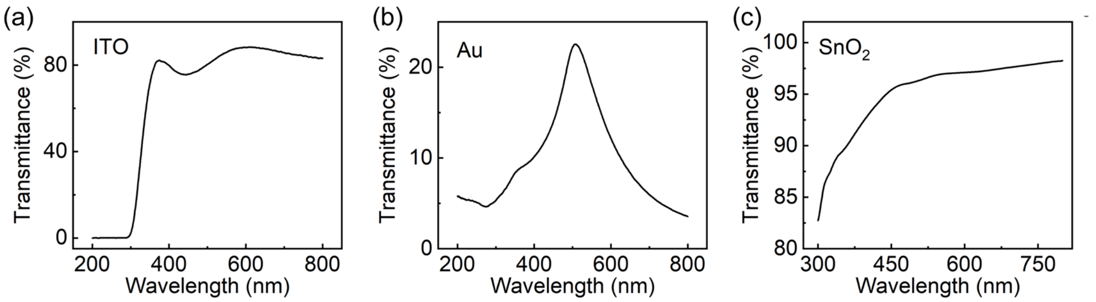

Additional file 1: Fig.S5 The light transmittance of ITO (a), 80 nm Au film by thermal evaporation (b), and SnO_2 thin film (c).

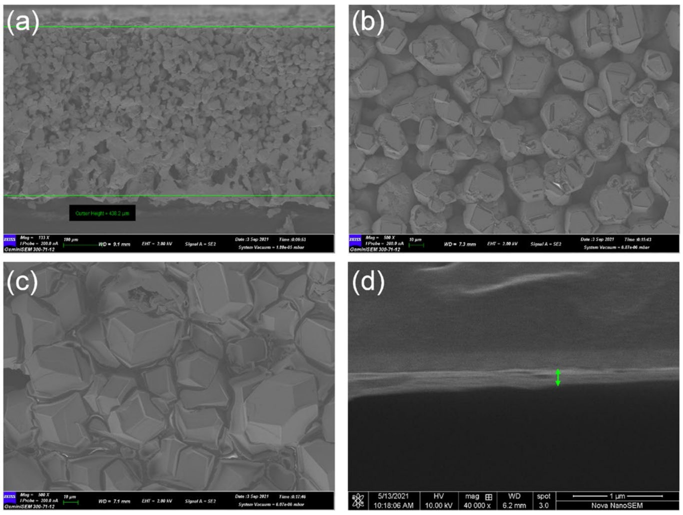

Additional file 1: Fig.S6 Cross-sectional (a), bottom surface (b), and top surface (c) scanning electron microscope (SEM) images of $MAPbI_3$ thick film. Cross-sectional SEM image of SnO_2 thin film.
